# Evaluation of the susceptibility and fatality of lung cancer patients towards the COVID-19 infection: A systemic approach through analyzing the ACE2, CXCL10 and their co-expressed genes

**DOI:** 10.1016/j.crmicr.2021.100022

**Published:** 2021-02-09

**Authors:** Tousif Bin Mahmood, Afrin Sultana Chowdhury, Mohammad Uzzal Hossain, Mehedee Hasan, Shagufta Mizan, Md. Mezbah-Ul-Islam Aakil, Mohammad Imran Hossan

**Affiliations:** a Department of Biotechnology and Genetic Engineering, Noakhali Science and Technology University, Noakhali 3814, Bangladesh; b Bioinformatics Division, National Institute of Biotechnology, Dhaka 1349, Bangladesh; c Department of Genetic Engineering and Biotechnology, University of Chittagong, Chittagong 4331, Bangladesh

**Keywords:** COVID-19, SARS-CoV-2, Lung cancer, ACE2, CXCL10, Biomarker

## Abstract

Coronavirus disease-2019 (COVID-19) is a recent world pandemic disease that is caused by a newly discovered strain of the Severe Acute Respiratory Syndrome Coronavirus-2 (SARS- CoV-2). Patients with comorbidities are most vulnerable to this disease. Therefore, cancer patients are reported to be more susceptible to COVID-19 infection, particularly lung cancer patients. To evaluate the probable reasons behind the excessive susceptibility and fatality of lung cancer patients to COVID-19 infection, we targeted the two most crucial agents, Angiotensin-converting enzyme 2 (ACE2) and C-X-C motif 10 (CXCL10). ACE2 is a receptor protein that plays a vital role in the entry of SARS-CoV-2 into the host cell and CXCL10 is a cytokine mainly responsible for the lung cell damage involving in a cytokine storm. By using the UALCAN and GEPIA2 databases, we observed that ACE2 and CXCL10 are mostly overexpressed in lung adenocarcinoma (LUAD) and lung squamous cell carcinoma (LUSC). We then identified the functional significance of ACE2 and CXCL10 in lung cancer development by determining the genetic alteration frequency in their amino acid sequences using the cBioPortal web portal. Lastly, we did the pathological assessment of targeted genes using the PANTHER database. Here, we found that ACE2 and CXCL10 along with their commonly co-expressed genes are involved respectively in the binding activity and immune responses in case of lung cancer and COVID-19 infection. Finally, based on this systemic analysis, we concluded that ACE2 and CXCL10 are two possible biomarkers responsible for the higher susceptibility and fatality of lung cancer patients towards the COVID-19.

## Highlights

• The expression of ACE2 and CXCL10 is upregulated in lung cancer.
• 64 and 6 mutations were identified in ACE2 and CXCL10 protein sequences, respectively.
• ACE2 and CXCL10 are found as the hub proteins in the PPI network of COVID-19 development.
• 803 co-expressed genes of ACE2 are found to be involved in binding activity.
• 68 co-expressed genes of CXCL10 are identified involving in the immune response.

## 1 Introduction

Zoonotic diseases have been a significant concern of public health in the previous decades. Pandemics like Influenza, London plague, smallpox, Spanish flu, AIDS majorly stand examples of cross-species transmission (CST) in several viruses and their fatality rates impose the severity of zoonotic diseases in global populations (Faria et al., 2013, Shereen et al., 2020, Spinelli, 2020). Similar to previous epidemics and pandemics, Severe acute respiratory syndrome coronavirus 2 (SARS-CoV-2), the causative agent of Coronavirus Disease 2019 (COVID-19) is in no way behind in the race of body counts, which to date stands at a 102,635,329 infections and 2,216,420 deaths as of the record of Coronavirus Resource Center of John Hopkins University (JHU) (Dong et al., 2020, Jennifer, 2020). Although vaccines against COVID-19 have already been formulated, developed and being administered, COVID-19 still requires attention in terms of its clinical outcomes in patients with comorbidities as well as those who are immunocompromised.

When it comes to analyzing risk groups, initial studies on COVID-19 have pinpointed that people with comorbidities like diabetes, hypertension, carcinoma, cardiovascular disease, chronic kidney disease are more susceptible to SARS-CoV-2 infections as well as being exposed to a more severe prognosis of COVID-19 (Zhou et al., 2020, Yang et al., 2020). However, within this risk cohort, people with cancer are comparatively more vulnerable to COVID-19 rather than people having other underlying clinical complications due to having a more suppressed and compromised immunity as well as not being able to access the required follow-up therapeutic procedures due to the restrictions imposed because of COVID-19 (Saini et al., 2020).

As COVID-19 is a disease of the respiratory tract, previous studies have concluded that patients with lung cancer often experience severe manifestations when infected by SARS-CoV-2 and are at a higher mortality risk than patients with other cancer types (Antonio et al., 2020). An investigation that took place in China suggested that 18 out of 1590 cases of COVID-19 patients had a cancer history. Among these 18 patients, 5 patients had a history of lung cancer (28%). Another multi-cancer study in New York implied that 22 lung cancer patients (21%) were identified from 105 COVID-19 infected cancer patients (Dai et al., 2020). In both of the cases, lung cancer was found as the most frequent cancer type. In terms of fatality rate, a recent study in New York provides information that 6 out of 11 (55%) lung cancer patients died when infected by COVID-19 (Mehta et al., 2020, Landman et al., 2020).

If we scrutinize the overall pathophysiology of COVID-19, we will find that two proteins play a crucial role in the progression of the disease. The first one being the angiotensin-converting enzyme-2 (ACE2) receptor which mediates the SARS-CoV-2 virus's entry inside the host by letting the virus anchor using its envelope (Indwiani Astuti, 2020, Guo et al., 2020, Xu et al., 2020); the second once being cytokines which hallmark the entry of the pathogen by elucidating inflammatory signals. Previous studies have stated that the cytokine storm exacerbates the manifestations of SARS-CoV-2 inside the host through a sudden spike in its levels, triggering immune cells to rush in the site of inflammation and cause extensive tissue damage leading to subsequent development of Acute Respiratory Distress Syndrome (ARDS), multi-organ failure and ultimately death (Abassi et al., 2020, Routley, 2020, Barnes et al., 2020). Although at an initial stage the activated macrophages trigger the release of cytokines to the injured lung cells for resolving the inflammation, the release of pro-inflammatory signaling molecules once again increases the cytokine levels (Abassi et al., 2020, Routley, 2020, Barnes et al., 2020, Huang et al., 2020). These severe manifestations are amplified in patients with lung cancer, due to the preexisting elevated levels of ACE2 receptor, which facilitates the entry and localization of SARS-CoV-2 and cytokines (Feng et al., 2010, Zhang et al., 2020a,b, Qian et al., 2020). Recent reports claimed that C-X-C motif 10 (CXCL10), also known as Interferon gamma-induced protein 10 (IP-10) is one of the most crucial cytokines associated with COVID-19 disease severity (Yang et al., 2020). Moreover, studies have also drawn a conclusion to the fact that the CXCL10 pro-inflammatory chemokine is also associated with pathological processes like infectious diseases as well as cancers and cause significant tissue damage (Kanda et al., 2007, Lee et al., 2009). Hence, by analyzing both the ACE2 receptor and the CXCL10 chemokine in terms of expression, the nature of COVID-19 in lung cancer patients can be highlighted and understood at a comprehensive level and questions- why a certain cancer cohort is more susceptible to COVID-19 can be addressed.

In this *in silico* study, we analyzed ACE2 and CXCL10 in terms of expression pattern, functional characterization and mutation in lung adenocarcinoma (LUAD) and lung squamous cell carcinoma (LUSC) to assess their possible involvement in the severe prognosis of COVID-19 in patients with lung cancer. We utilized existing cancer and expression databases and adapted protein-protein interaction network (PPIN) analysis schemas which currently is an effective method for scrutinizing the role of different proteins and genes in diseases and disorders at a molecular level. Because our analysis involved ACE2, CXCL10 and their coexpressed genes with regards to both healthy and lung cancer individuals, a clear comparison of the cohorts can help identify specific causes of COVID-19’s clinical outcomes in lung cancer patients in regards to ACE2 and CXCL10 and can help in the proper management of COVID-19 in this cohort.

## 2 Materials and methodology

### 2.1 Expression analysis of the targeted genes

#### 2.1.1 Analysis of gene expression in lung cancer

Data corresponding to ACE2 and CXCL10 mRNA expression patterns in lung cancer were retrieved from Tumor Immune Estimation Resource (TIMER) (http://cistrome.org/TIMER/). This tool allows a comprehensive analysis of the immune-suppressive nature of diverse cancer types. This web resource consists of six principal catalogs of analysis which allow to analyse gene expression data and their interconnections along with the immune-suppressed cancer cells (Li et al., 2017).

#### 2.1.2 Extensive analysis of gene expression pattern in LUAD and LUSC

In-depth expression analysis of ACE2 and CXCL10 in LUAD and LUSC was done by using the TCGA data from the UALCAN web portal (http://ualcan.path.uab.edu/). This is a publicly accessible web platform that is frequently used to analyze the expression, co-expression of multiple genes, and their association with the clinical prognosis of variant cancer types by utilizing the TCGA data. UALCAN provides analytical data for 31 types of cancer (Chandrashekar et al., 2017).

#### 2.1.3 Comparative analysis of the expression of targeted genes in LUAD and LUSC

Gene expression profiling interactive analysis (GEPIA) 2 database (http://gepia2.cancer-pku.cn/#index) is a web portal to analyze the mRNA expression of 8587 normal and 9736 tumor samples using the TCGA data. Besides the expression analysis, this database is also used for the gene-specific comparative analysis of different cancer types (Tang et al., 2019). Therefore, investigating the difference between ACE2 and CXCL10 expression in LUAD and LUSC was carried out by using the GEPIA 2 web portal.

### 2.2 Functional characterization of the targeted genes

#### 2.2.1 Mutation and CNAs determination in the targeted proteins

Exploration of significant genetic changes in ACE2 and CXCL10 was done by using cBioPortal (https://www.cbioportal.org/). This software is an interactive web portal for systematic analysis of the multidisciplinary data sets of cancer genomics. Providing the data from more than 5000 tumor samples, this database is mostly used for the molecular profiling of cell lines and cancer tissue, mapping the frequency of mutations and other genetic alterations utilizing multidimensional cancer studies (Cerami et al., 2012).

#### 2.2.2 Construction of protein-protein interaction network

Genes that are involved in COVID-19 development and predicted to have a dominant association with the viral disease were retrieved from the Comparative Toxicogenomics Database (CTD) (http://ctdbase.org/). CTD is a regularly updated database that delivers readily understandable records representing the gene-disease relationships (Mattingly et al., 2006). To analyze the interconnection between the proteins significantly associated with COVID-19, a network was generated among them by using the STRING software (https://string-db.org/). This software is mainly used to construct a protein-protein interaction network for showing multi-variant associations among the targeted proteins (Szklarczyk et al., 2017).

### 2.3 Co-expression analysis of the targeted genes

#### 2.3.1 Identification of the co-expressed genes

The genes co-altered with the expression of ACE2 and CXCL10 in lung cancer were identified by using the R2: Genomics and Visualization platform (https://hgserver1.amc.nl/cgi-bin/r2/main.cgi). This database is an enriched resource of gene-specific datasets that allows to analyze, interpret, and reveal prominent outcomes of clinical research studies (Koster and Versteeg, 2008).

#### 2.3.2 Determination of commonly co-expressed genes

The commonly co-altered genes of ACE2 and CXCL10 in both lung cancer and COVID-19 are determined by constructing the Venn diagrams using the Bioinformatics and Evolutionary Genomics (http://bioinformatics.psb.ugent.be/webtools/Venn/) web portal. The Venn diagram can generate a graphical output that represents the common elements of each listed intersection (Larran and Saeys, 2007).

### 2.4 Interpretation of the functional role of the targeted genes

**Fig. 1:**
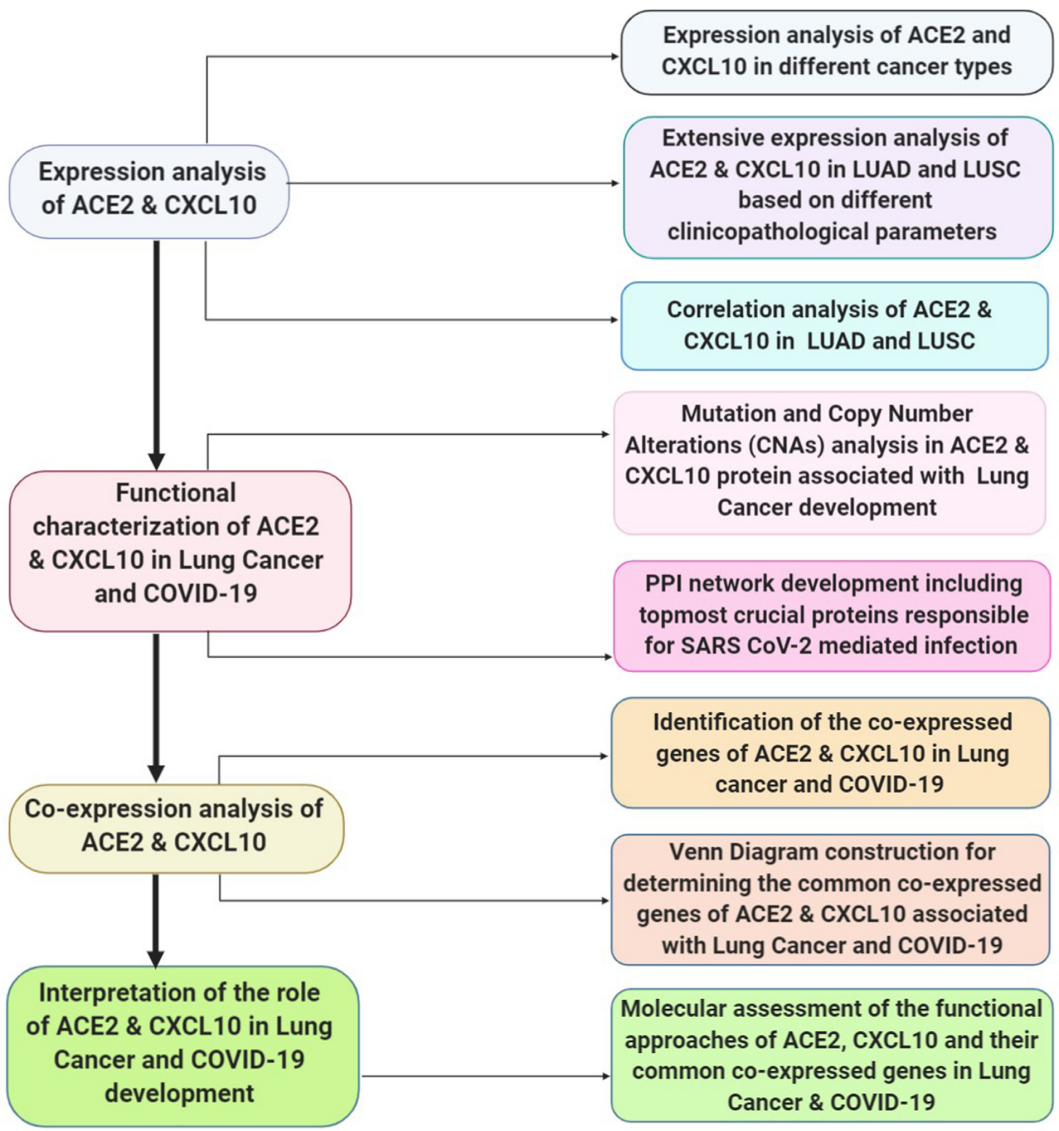
A schematic diagram representing the overall workflow of the study.

An integrative molecular assessment of the functional approaches of ACE2 and CXCL10 associated with the lung cancer and fatal type of COVID-19 development was attributed by using Protein Analysis Through Evolutionary Relationships (PANTHER) (http://www.pantherdb.org/) tool. This is a comprehensive tool that can classify genes in terms of various attributes such as biological procedures, cellular structures, molecular attitudes, and pathways (Mi et al., 2013, Rahman et al., 2020) (Fig. 1).

## 3 Results

### 3.1 Expression of ACE2 and CXCL10 in lung cancer

**Fig. 2:**
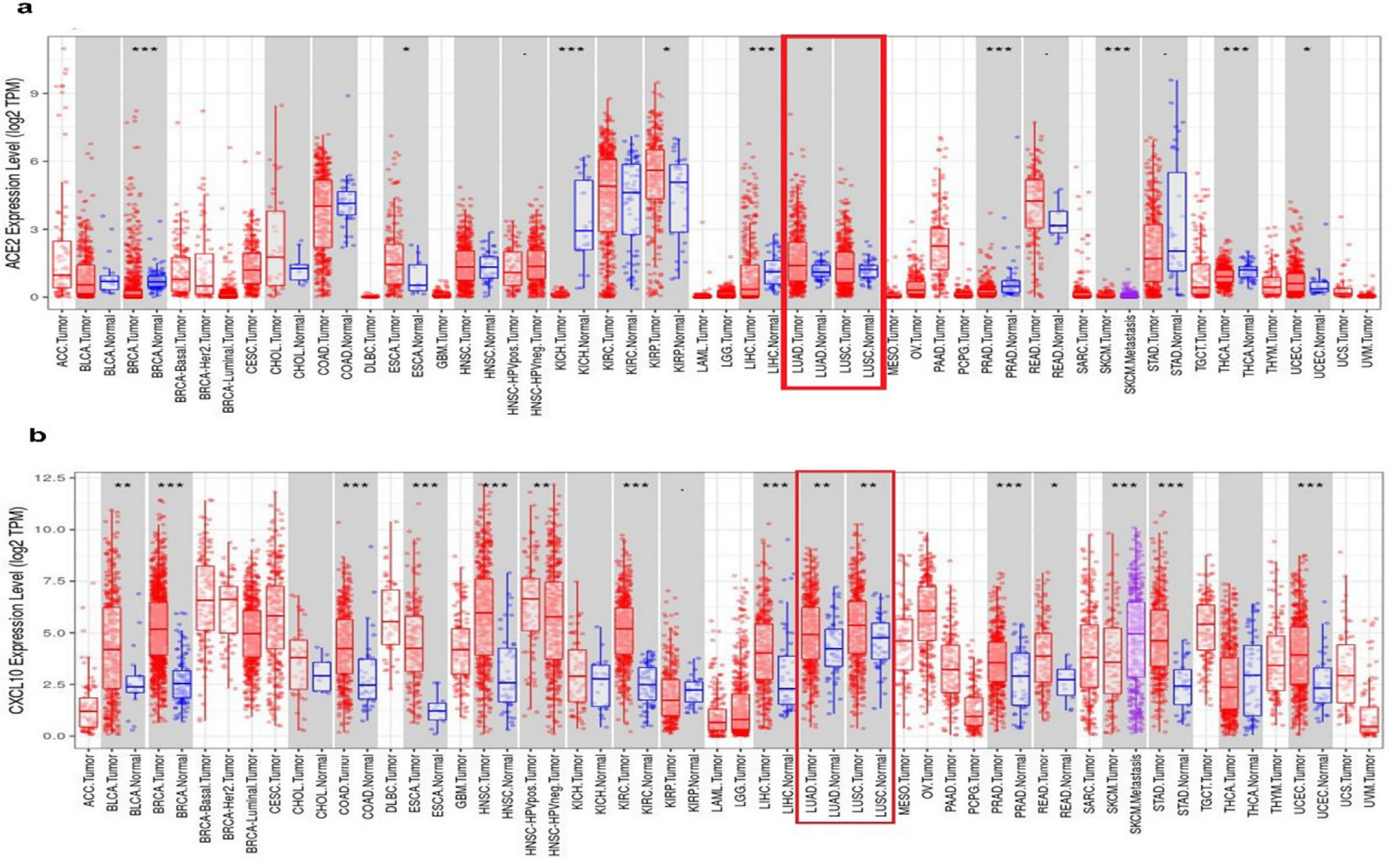
The expression analysis of ACE2 and CXCL10 by using TIMER **(a)** Different level of ACE2 mRNA expression is shown in multiple cancer studies whereas the overexpression of ACE2 in LUAD and LUSC is marked in the red box **(b)** Different level of CXCL10 mRNA expression is shown in multiple cancer studies whereas the overexpression of CXCL10 in LUAD and LUSC is marked in the red box. *P-*value codes: *<0.05, **<0.01, ***<0.001 Abbreviation: LUAD- Lung Adenocarcinoma; LUSC- Lung squamous cell carcinoma

Firstly, we analyzed the different expression patterns of ACE2 and CXCL10 in multidisciplinary cancer types using the TIMER database. Here, we found that ACE2 mRNA expression was significantly upregulated in lung adenocarcinoma (LUAD) (Fig. 2a). Also an increased level of expression in both LUAD and LUSC was shown by CXCL10 when compared to its normal tissues. *p*-value < 0.01 was evidenced in both the cancer types for CXCL10 (Fig. 2b). Overall, these results gave an indication of the overexpression of ACE2 and CXCL10 in LUAD and LUSC.

### 3.2 Analysis of ACE2 and CXCL10 expression pattern in LUAD and LUSC

**Fig. 3:**
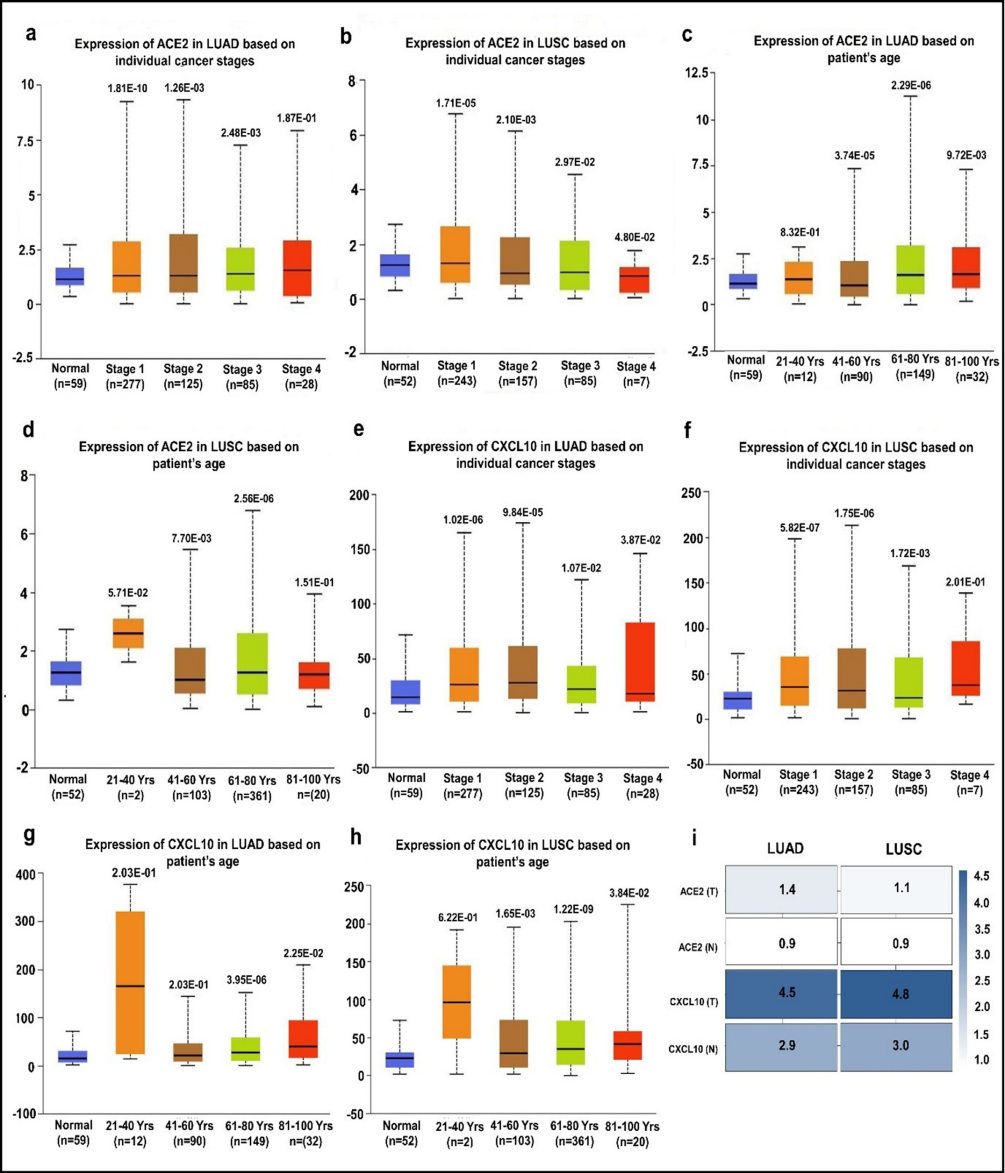
Expression analysis of ACE2 and CXCL10 in lung cancer using the UALCAN database; (**a**) ACE2 gene expression in LUAD based on different cancer stages; (**b**) ACE2 gene expression in LUSC based on different cancer stages; (**c)** ACE2 gene expression in LUAD based on different age groups; (**d)** ACE2 gene expression in LUSC based on different age groups; (**e**) CXCL10 gene expression in LUAD based on different cancer stages (**f**) Expression level of CXCL10 gene in LUSC based on different cancer stages; (**g**) CXCL10 gene expression in LUAD based on different age groups; (**h**) CXCL10 gene expression in LUSC based on different age groups; (**i**) Comparative analysis of ACE2 and CXCL10 gene expression in LUAD and LUSC based on the expression score in tumor tissues (T) and normal tissues (N).

We performed an extensive analysis to evaluate the association of ACE2 and CXCL10 expression with multiple clinicopathological parameters using the TCGA dataset retrieved from the UALCAN data mining platform. Here, we found an overall upregulated expression pattern of ACE2 in LUAD and LUSC compared to the normal condition based on individual cancer stages and different age groups. In terms of LUAD, the ACE2 expression level was found higher in all of the cancer stages where stage 4 shows the most elevated level compared to other cancer stages (Fig. 3a). Regarding LUSC, we found an increased level of ACE2 in all of the cancer stages except stage 4 (Fig. 3b). However, a similar kind of outcome representing the overexpression of ACE2 was observed in terms of variant age groups of the LUAD and LUSC patients. Among these age groups, ACE2 expression is most upregulated in the case of 61–80 years old patients. However, a slightly down-regulated expression of ACE2 was found for the age group of 41–60 years (Fig. 3c and d).

Next, we investigated the changes of CXCL10 expression based on different clinicopathological parameters. Here, we observed significant upregulation in CXCL10 expression in all cancer stages for both LUAD and LUSC patients. Among the four different cancer stages, the topmost elevated expression of CXCL10 was found in stage 2 for LUAD patients and stage 4 of LUSC patients (Fig. 3e and f). Besides the different cancer stages, we also found an elevated level of CXCL10 expression based on different age groups of LUAD and LUSC patients. Interestingly, CXCL10 expression was most upregulated in the younger age groups (21–40 years) compared to all other age groups of LUAD and LUSC (Fig. 3g and h).

After that, we generated an interactive heat map by using GEPIA 2 which compared the expression level of ACE2 and CXCL10 along with both cancer types, LUAD, and LUSC. This comparative illustration is represented based on the expression score of ACE2 and CXCL10. Both the genes showed a higher expression level in the tumor cells compared to the normal cells. However, ACE2 showed a higher expression score (1.4) in LUAD rather than the score (1.1) in LUSC. On the counterparts, CXCL10 showed a higher expression score (4.8) in LUSC than the score (4.5) in LUAD (Fig. 3i).

### 3.3 Analysis of genetic changes in ACE2 and CXCL10 protein sequences associated with lung cancer development

**Fig. 4:**
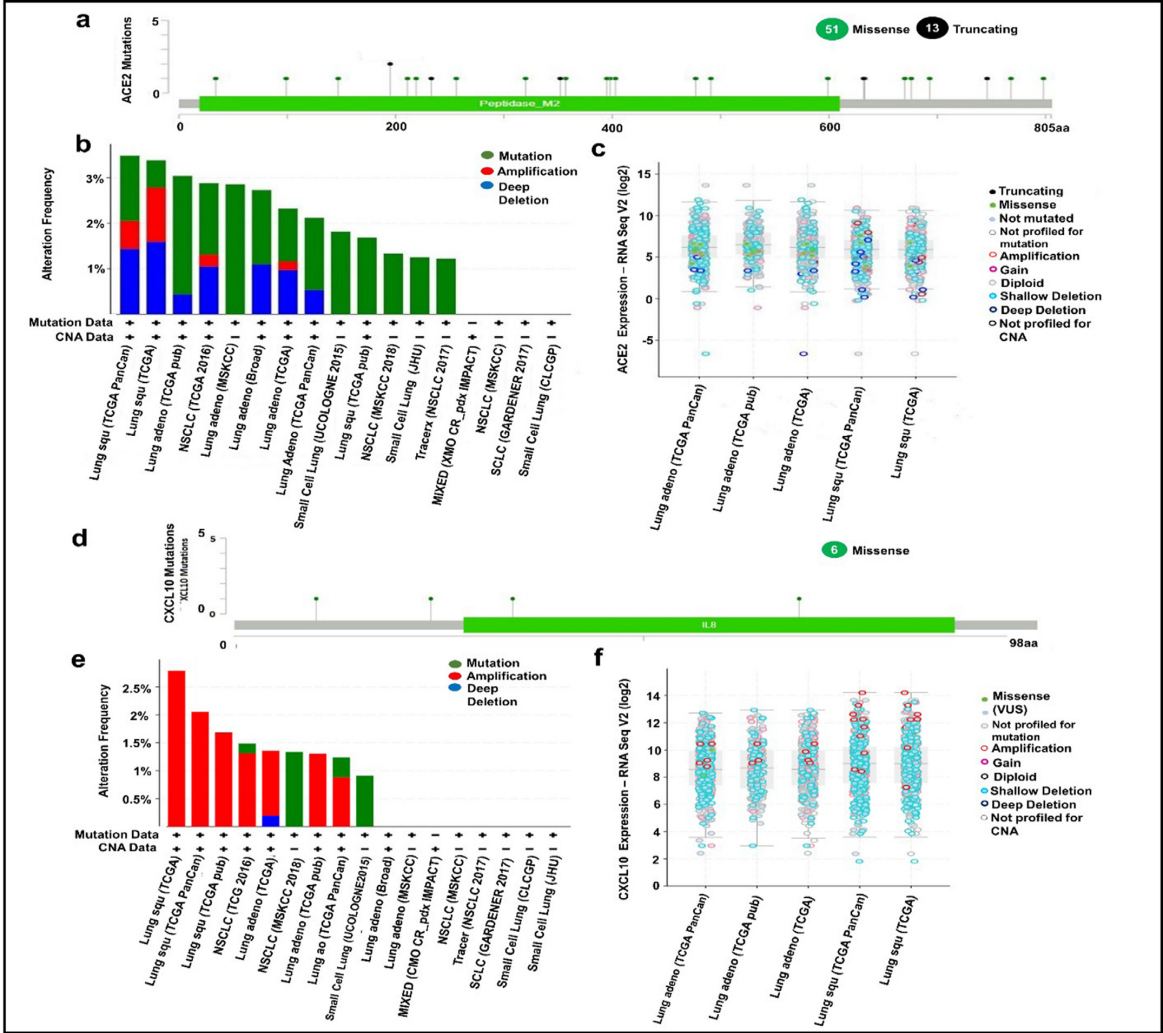
The Functional characterization of ACE2 and CXCL10 in lung cancer development by using the cBioPortal (**a**) 64 Mutations in ACE2 protein sequence was figured out by using lollipop plots; (**b**) Three types of alteration frequencies of ACE2 in lung cancer were presented in bar diagram; (**c**) The expression level of differently categorized genetic alterations was presented for ACE2; (**d**) Total 6 mutation in CXCL10 protein was presented by lollipop plots. (**e**) Three variant types of alteration frequency in CXCL10 were presented in the bar diagrams. (**f**) The expression level regarding the multiple categories of genetic alteration was represented in graphical plots based on the log scale (RNA seqV2).

To evaluate the functional significance of ACE2 and CXCL10 in lung cancer development, we generated data representing multiple genetic alterations in ACE2 and CXCL10 mRNA using the cBioPortal database. Firstly, we prepared a query for ACE2 in this database using 6075 samples of 5719 lung cancer patients from 21 studies. From this analysis, we found out 64 mutations at 24 different locations of the 805 amino acids long human ACE2 protein (Table 1). Out of these 64 mutations, 51 were missense type, while the other 13 were recognized as truncated type (Fig 4a). After that, we extended our analysis to explore the frequency of genetic alterations in the ACE2 gene by using data from different lung cancer studies. Through this analysis, we observed that ACE2 is mostly altered in LUSC ranging the highest frequency of 3.49%. Though the alteration frequency fluctuated variously in multiple types of lung cancer studies, we managed to reveal that the least rate of alteration occurs in small cell lung cancer (Fig. 4b). Then, we focused on the expression level analysis of unique types of genetic alteration. In this case, we found that the highest level of copy number alteration is occurred due to the shallow deletion type of genetic alteration. The second most significant genetic change is done by amplification type of copy number alteration. Overall, variant natures of genetic alteration in ACE2 are assembled to contribute to lung cancer development (Fig. 4c).

**Table 1:** A list of genetic alternations in ACE2 protein sequences associated with lung cancer development.

Cancer study	Sample size	Protein change	Mutation type	Sample ID
		G747Wfs*27	FS ins	LUAD-B00416
Lung Adenocarcinoma (Broad, Cell	183	R768L	Missense	LUAD_E00522
2012)		R357	Missense	LUAD-LIP77
		I256M	Missense	TCGA-95-7948-01
		R219P	Missense	TCGA-44-2659-01
Lung Adenocarcinoma (TCGA,	586	G211W	Missense	TCGA-44-7670-01
Firehose Legacy)		L320F	Missense	TCGA-69-7980-01
		D693N	Missense	TCGA-73-4658-01
		H34N	Missense	TCGA-99-7458-01
Lung Adenocarcinoma (MSKCC, Science 2015)	35	N599K	Missense	FR9547
		I256M	Missense	TCGA-95-7948-01
		R219P	Missense	TCGA-44-2659-01
		G211W	Missense	TCGA-44-7670-01
Lung Adenocarcinoma (TCGA,	566	L320F	Missense	TCGA-69-7980-01
PanCancer Atlas)		D693N	Missense	TCGA-73-4658-01
		H34N	Missense	TCGA-99-7458-01
		T798P	Missense	TCGA-55-8205-01
		V670L	Missense	TCGA-55-8506-01
		A99S	Missense	TCGA-62-A46R-01
		I256M	Missense	TCGA-95-7948-01
		R219P	Missense	TCGA-44-2659-01
Lung Adenocarcinoma (TCGA,	230	G211W	Missense	TCGA-44-7670-01
Nature 2014)		L320F	Missense	TCGA-69-7980-01
		D693	Missense	TCGA-73-4658-01
		H34N	Missense	TCGA-99-7458-01
Lung Squamous Cell Carcinoma	511	V491L	Missense	TCGA-22-1016-01
(TCGA, Firehose Legacy)		G147V	Missense	TCGA-39-5035-01
		X233_splice	Splice	TCGA-43-6770-01
		V491L	Missense	TCGA-22-1016-01
		G147V	Missense	TCGA-39-5035-01
		X233_splice	Splice	TCGA-43-6770-01
Lung Squamous Cell Carcinoma	487	Y633Lfs*2	FS ins	TCGA-33-4587-01
(TCGA, PanCancer Atlas)		G395V	Missense	TCGA-56-7731-01
		W477R	Missense	TCGA-92-7341-01
		X195_splice	Splice	TCGA-L3-A524-01
		V491L	Missense	TCGA-22-1016-01
Lung Squamous Cell Carcinoma	178	G147V	Missense	TCGA-39-5035-01
(TCGA, Nature 2012)		X233_splice	Splice	TCGA-43-6770-01
Non-Small Cell Lung Cancer (MSK, Cancer Cell 2018)	75	X195_splice	Splice	nsclc_mskcc_2018s50
		I256M	Missense	TCGA-95-7948-01
		G747Wfs*27	FS ins	LUAD-B00416-Tumor
		R768L	Missense	LUAD_E00522-Tumor
		R219P	Missense	TCGA-44-2659-01
		G211W	Missense	TCGA-44-7670-01
		L320F	Missense	TCGA-69-7980-01
		H34N	Missense	TCGA-99-7458-01
		V491L	Missense	TCGA-22-1016-01
		G147V	Missense	TCGA-39-5035-01
		X233_splice	Splice	TCGA-43-6770-01
Pan-Lung Cancer (TCGA, Nat	1144	Y633Lfs*2	FS ins	TCGA-33-4587-01
Genet 2016)		T798P	Missense	TCGA-55-8205-01
		V670L	Missense	TCGA-55-8506-01
		G395	Missense	TCGA-56-7731-01
		A99S	Missense	TCGA-62-A46R-01
		W477R	Missense	TCGA-92-7341-01
		X195_splice	Splice	TCGA-L3-A524-01
		R357S	Missense	LUAD-LIP77-Tumor
		A403V	Missense	CRUK0001-R1
Non-Small Cell Lung Cancer	447	A403V	Missense	CRUK0001-R2
(TRACERx, NEJM & Nature 2017)		A403V	Missense	CRUK0001-R3
		K676E	Missense	CRUK0027-R2
		E398K	Missense	585208
Small Cell Lung Cancer (Johns	80	X632_splice	Splice	sclc_ucologne_2015_S02093
Hopkins, Nat Genet 2012)		K353Rfs*12	FS del	sclc_ucologne_2015_S02277

Next, we went through a similar type of analysis for interpreting the genetic alterations in the CXCL10 mRNA regarding lung cancer. In this instance, we pointed out 6 missense type of mutations with a somatic mutation frequency of 0.1% where 2 duplicate mutations were present (Table 2). The mutations are evidenced at 4 different locations of the 98 amino acids long CXCL10 protein sequence (Fig. 4d). We then analyze genetic alteration frequency in the CXCL10 mRNA distinct numbers of lung cancer studies. In this regard, we observed the highest rate of alteration frequency (2.79%) in CXCL10 mRNA for LUSC. Interestingly, amplification is the only type of alteration in this case. However, for other cases, fluctuation in alteration frequency and changes in the nature of alteration was observed. For instance, the TCGA data of LUSC represented alteration frequency only for amplification type whereas UCOLOGENE 2015 revealed that alterations in CXCL10 of small cell lung cancer are completely mediated by mutation type genetic change (Fig. 4e). Last of all, we did an extensive analysis of the different expression levels of unique alteration types in CXCL10 mRNA. From this analysis, we experienced that amplification is the most upregulated copy number alteration considering their level of expression while shallow deletion is the most common type of alteration considering their expression frequency (Fig. 4f). Overall, the functional characterization of ACE2 and CXCL10 by using multiple lung cancer studies provides some of the vital evidence of their intimate relationship with lung cancer development.

**Table 2:** A list of genetic alternations in ACE2 protein sequences associated with lung cancer development.

Cancer study	Sample size	Protein change	Mutation type	Sample ID
Pan-Lung Cancer (TCGA, Nat	1144	S34I	Missense	TCGA-L9-A7SV-01
Genet 2016)		K69N	Missense	TCGA-NJ-A4YP-01
Lung Adenocarcinoma (TCGA,	566	S34I	Missense	TCGA-L9-A7SV-01
PanCancer Atlas)		K69N	Missense	TCGA-NJ-A4YP-01
Non-Small Cell Lung Cancer (MSK, Cancer Cell 2018)	75	C10F	Missense	nsclc_mskcc_2018s29
Small Cell Lung Cancer (U Cologne, Nature 2015)	120	L24V	Missense	sclc_ucologne_2015_S02322

### 3.4 Determination of ACE2 and CXCL10 assisted PPI network associated with COVID-19 development

**Fig. 5:**
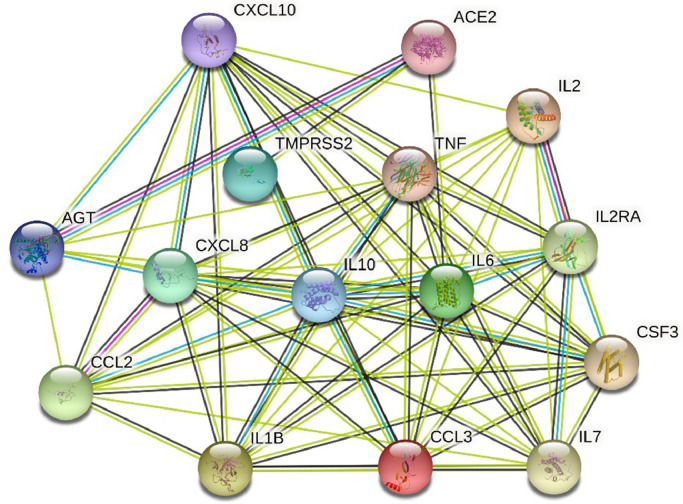
A protein-protein interaction network representing the interconnection of the functional proteins associated with COVID-19 infection. ACE2 is mainly interconnected with AGT, TMPRSS2, and IL1B whereas CXCL10 is interlinked with other cytokines such as IL-6, IL-7, TNF, and IL-10. Many other cross-linked interconnections are also found for other proteins. Abbreviation: CXCL10 – C-X-C motif 10; ACE2- Angiotensin converting enzyme 2; AGT- Angiotensin; CXCL8- C-X-C motif 8; IL6- Interleukin 6; TMPRSS2- Transmembrane protease, serine 2; IL2- Interleukin 2; IL2RA- Interleukin 2 receptor alpha chain; CCL2- C-C motif ligand 2; TNF- Tumor necrosis factor; IL10- Interleukin 10; CSF3- Colony stimulating factor; IL1B- Interleukin 1 beta; CCL3- C-C motif ligand 3; IL7- Interleukin 7

Multiple numbers of genes are responsible directly or indirectly for COVID-19 development. Using the Comparative Toxicogenomics Database (CTD), we managed to identify 12,347 genes associated with COVID-19 disease based on their inference score. Each of these genes has either curated association to the disease or an inferred association *via* a curated chemical interaction. Extracting the huge data set, 15 curated genes were identified as the biomarkers or therapeutic targets for COVID-19 treatment in which ACE2 and CXCL10 were included (Table 3). By utilizing the translated protein sequences of these 15 genes a PPI network was constructed through the STRING database (Fig. 5). Following this, we found out 74 connecting edges among the selected proteins, though the predicted edges were only 16 according to the information provided by the database itself. That means the network represents more interconnections than the expected outcome. Such affluence indicates that the proteins are functionally connected, as a group. We also found that ACE2 and CXCL10 proteins are interconnected along with other protein components associated with COVID-19 development. It is clear evidence that ACE2 and CXCL10 are crucial proteins contributing to the COVID-19 disease progression.

**Table 3:** The List of curated genes directly associated with COVID-19 development.

Name of gene	Curated association	Inference score
CCL2	Biomarker	30.22
TNF	Biomarker	28.55
IL6	Biomarker	25.40
IL10	Biomarker	25.01
IL1B	Biomarker	21.27
IL8	Biomarker	20.75
AGT	Biomarker	18.01
IL2	Biomarker	15.73
CCL3	Biomarker	11.73
CXCL10	Biomarker	11.46
ACE2	Biomarker and Therapeutic Target	9.16
TMPRSS2	Biomarker	6.38
IL2	Biomarker	6.19
IL2RA	Biomarker	3.20
CSF3	Biomarker	2.28

### 3.5 Estimation of the commonly co-expressed genes of ACE2 and CXCL10 associated with lung cancer and COVID-19 development

**Fig. 6:**
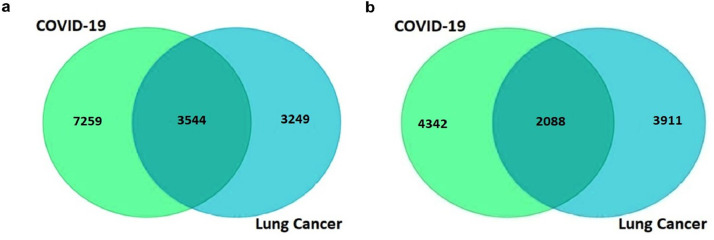
Graphical representation of commonly co-expressed genes of ACE2 and CXCL10 in lung cancer and COVID-19 **(a)** The Venn diagram represents 3544 co-expressed genes of ACE2 **(b)** The Venn diagram represents 2088 commonly co-expressed genes of CXCL10 associated with both of the diseases.

To identify the genes that are correlated with the expression of ACE2 and CXCL10, we went through a comprehensive analysis by using the R2: Genomics and Visualization web portal. From here, we explored the co-expressed genes of ACE2 and CXCL10 responsible for lung cancer and COVID-19 development by utilizing the TCGA data. We identified a total of 6793 co-expressed genes of the ACE2 associated with LUAD and LUSC whereas the number of co-expressed genes related to COVID-19 was 10803. Similarly, we determined 5999 genes that are co-altered with CXCL10 in the case of lung cancer development and 6430 co-expressed genes associated with COVID-19. A restriction of *p-*value < 0.01 was applied to each case of the analysis. After that, the lists representing co-expressed genes of ACE2 and CXCL10 in each of the cases of lung cancer and COVID-19 were utilized to construct Venn diagrams using the Bioinformatics and Evolutionary Genomics web tool. The two Venn diagrams revealed the commonly co-expressed genes of ACE2 and CXCL10 associated with lung cancer and COVID-19 development. In the case of ACE2, 3544 co-expressed genes were identified commonly associated with both of the disease conditions (Fig. 6a). On the other hand, 2088 genes were identified which are co-altered along with the expression of CXCL10 in both cases of lung cancer and COVID-19 (Fig. 6b).

### 3.6 Molecular assessment of the functional approaches of ACE2 and CXCL10

**Fig. 7:**
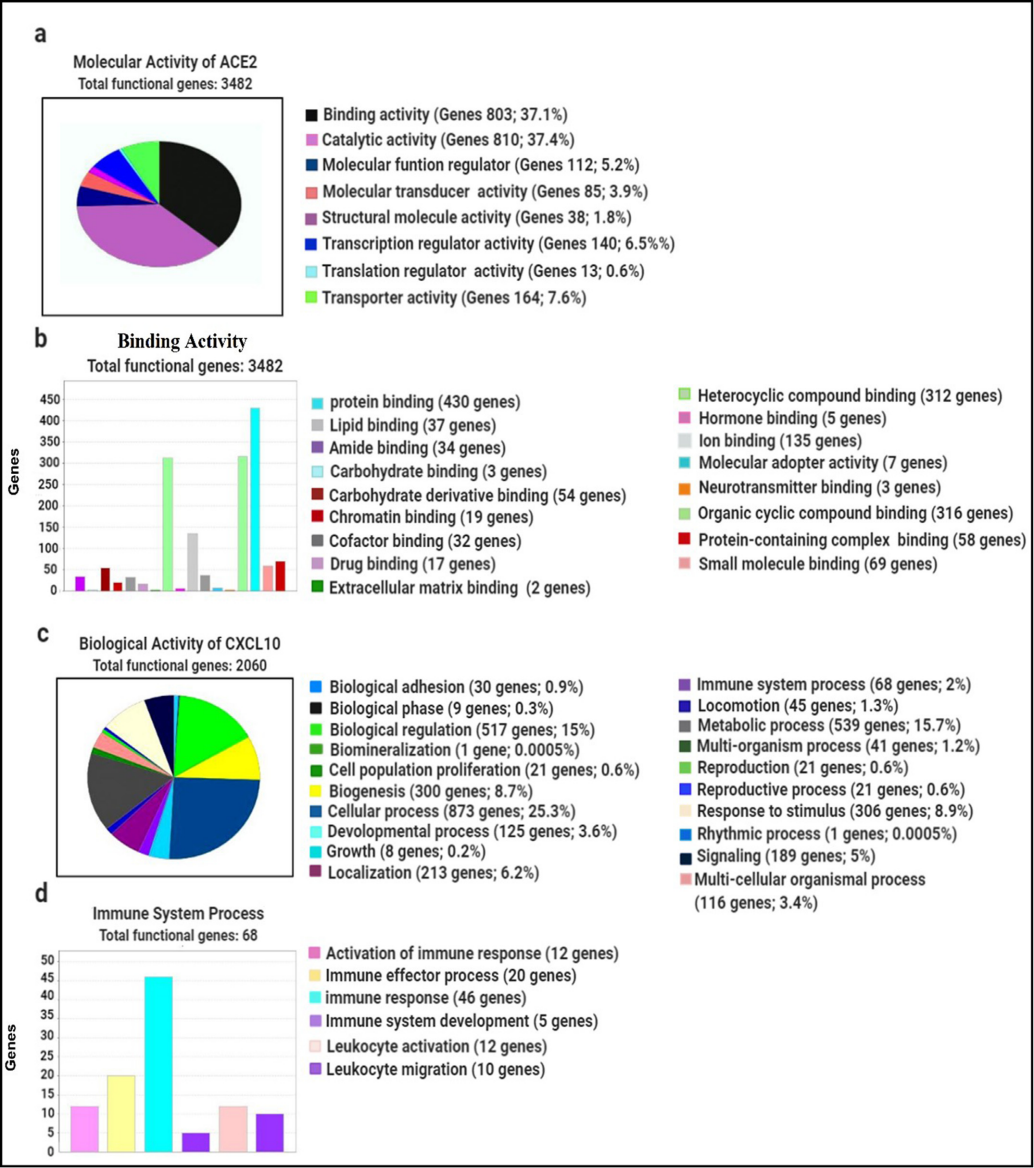
Evaluation of the functional attitudes of ACE2 and CXCL10 represented by using the PANTHER database **(a)** Eight classes of the molecular activity of ACE2 and its co-expressed genes were presented through a pie chart **(b)** In total 17 variant types of binding activities of 803 genes were represented by using a bar chart **(c)** Total 20 unique types of biological activities of CXCL10 and its co-expressed genes were represented using a pie chart **(d)** Six differently categorized immune system processes of the corresponding 68 co-expressed genes were presented through a bar chart.

To interpret the functional activity of ACE2 and CXCL10 in lung cancer and COVID-19, we used the lists of previously identified co-expressed genes using the PANTHER database. First of all, we processed a query for determining the molecular activity of ACE2 by listing the previously identified 3544 commonly co-expressed genes. We analyzed multiple types of molecular activity and found that a major portion (37.1%) of the genes (803) are involved in the binding activity (Fig. 7a). Through extended analysis, we observed the variant nature of the binding activities of corresponding 803 genes. Following this analysis, we observed that most of the genes are involved in protein binding activity (53.5%; 430 genes) (Fig. 7b).

After that, we looked forward to investigating the functional attitude of CXCL10 using the list of previously determined 2048 commonly co-expressed genes associated with lung cancer and COVID-19 development. By analyzing the biological processes, we observed that the listed genes are involved in performing a wide range of biological activities such as cellular process, biological regulation, metabolic process, immune system process, localization, biogenesis, etc. (Fig. 7c). However, by remarking our research aims, we performed an extended analysis with the co-expressed genes of CXCL10 involved in different immune processes. Regarding this analysis, we explored that 46 genes are involved in immune response, 5 genes act for immune system development, 12 genes are responsible for leukocyte activation whereas 10 genes are responsible for leukocyte migration, 12 genes contribute to the activation of immune response and 20 genes lead the immune effector process (Fig. 7d). Overall, many of the co-expressed genes of CXCL10 are found to play an important role in various branches of the immune system along with the functional activity of CXCL10.

## 4 Discussion

**Fig. 8:**
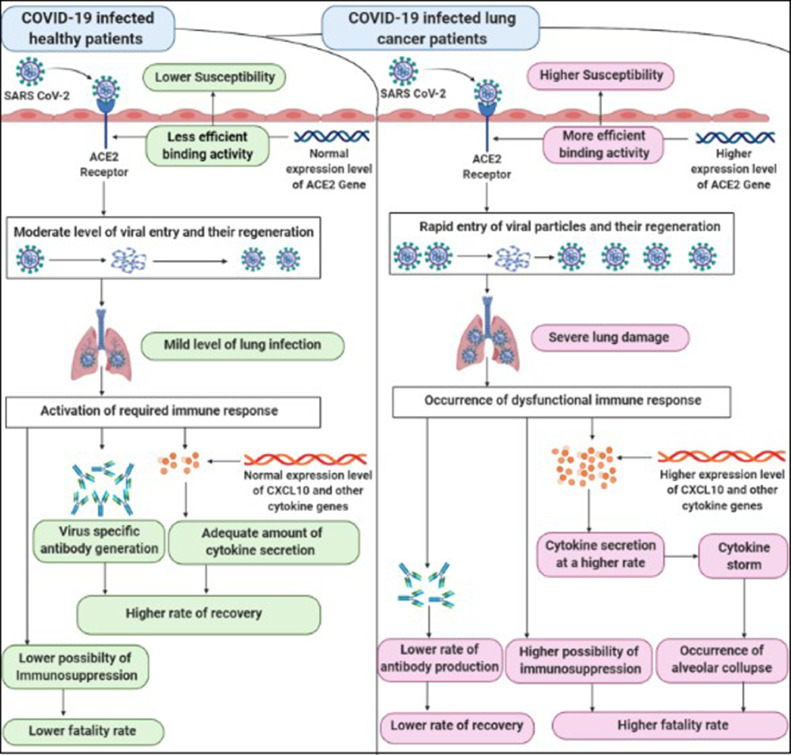
A comparative illustration representing the possible reasons for the higher susceptibility and fatality rate of the COVID-19 infected lung cancer patients rather than the COVID-19 infected healthy patients.

Because COVID-19’s clinical manifestations varied from cohort to cohort, it is quite important to address the key stakeholders for this variation for developing proper therapeutics against the disease as well as prevention and management in different cohorts (Landman et al., 2020, Vaninov, 2020). Previous studies have consistently held both ACE2 receptors as well as cytokine storms for the severe prognosis of COVID-19 and preexisting conditions like lung cancer just make the situation worse for the patients (Sidaway, 2020, Wan et al., 2020). Evidence that CXCL10 is one of the significant cytokines which is directly involved in the event of cytokine storm, tissue damage of the lung cell and subsequent development of Acute Respiratory Disease Syndrome (ARDS) indicates its importance as a biomarker for COVID-19 (Goh et al., 2020, Xu et al., 2020, Liu et al., 2020, Mehta et al., 2020). Based on these evidence, we analyzed the mRNA expression of ACE2 and CXCL10 in LUAD and LUSC attributing multidisciplinary parameters. We found that in almost all cases an over-expression of ACE2 in LUAD and LUSC was seen as compared to the cases with the normal conditions (Fig. 3a–d). Previous studies have reported the age groups exceeding 60 years as the most vulnerable to COVID-19 and our expression analysis of ACE2 and CXCL10 aligns with their finding. We observed that the most upregulated ACE2 expression was found for the age group 61-80 years which is reported as the topmost vulnerable age group for the COVID-19 infection (Liu et al., 2020). CXCL10 expression was found at an elevated level in both LUAD and LUSC (3e-h) which also indicates CXCL10’s involvement in lung carcinoma and the event of cytokine storm and subsequent disease fatality. We then applied a comparative analysis of ACE2 and CXCL10 expression in LUAD and LUSC based on their expression score by normal and tumor cells. In this case, we observed a higher expression score of these two genes in both LUAD and LUSC compared to the normal conditions (Fig. 3i). The results of these initial analyses provided primary evidence that overexpression of ACE2 receptor and enhanced CXCL10 cytokine expression might be the major possible reason for increased susceptibility and fatality of lung cancer patients towards COVID-19. Because overexpression of the ACE2 gene may result in increased binding activity of the ACE2 protein and therefore higher susceptibility towards the COVID-19 infection. On the other hand, the elevated expression of CXCL10 may also be involved in alveolar collapse by proceeding to the excessive secretion of cytokines (Fig. 8).

In regards to the aforementioned fact, we performed a functional assessment of ACE2 and CXCL10. From the Comparative Toxicogenomics Database (CTD), we retrieved the most significant 15 proteins which have a direct association with the COVID-19 disease development where ACE2 and CXCL10 proteins are also included (Table 3). Upon establishing a protein-protein interaction network (PPIN) among these proteins we found 74 nodes of interaction among the 15 proteins in which ACE2 and CXCL10 were predominantly explored as the hub proteins because the major portion of the nodes was assisted by these two proteins (Fig. 5). Previous studies on cancer have extensively used PPI network analysis methods to analyze causes based on a core protein in relation to other proteins by interpreting nodes and functionalities (Rezaei-Tavirani et al., 2017, Losuwannarak et al., 2020, Nibbe et al., 2010, Chuang et al., 2017, 2007). PPI networks have been a lucrative approach in the past years merely because they can answer queries relevant to the association of a particular protein with diseases at a molecular level. Moreover, PPI networks are equally helpful in contrasting expression levels of particular genes and proteins between normal cells and infected cells (Tang et al., 2011, Ideker et al., 2002, Hao et al., 2016, Dittrich et al., 2008). Our systematic analysis aligning to the general approach of PPIN analysis targeted two intricately important proteins ACE2 and CXCL10 involved in the cascade of biological processes triggered by the presence of a SARS-CoV-2 infection. Upon subjecting these two molecules, we observed that in both normal individuals and individuals with lung cancer, these two proteins are extensively involved in the overall pathophysiology and prognosis. This outcome provides extended evidence about the functional involvement and significant contribution of ACE2 and CXCL10 to the SARS-CoV-2 mediated infection development. Besides confirming the significance of ACE2 and CXCL10 in COVID-19 development, we also realized the need of determining their role in lung cancer development to establish a connection between lung cancer and COVID-19 through these targeted genes. Therefore, we performed a functional characterization of ACE2 and CXCL10 in lung cancer by analyzing the mutations and copy number alterations in their protein sequence based on 21 lung cancer studies. In total, 64 mutations at 24 different locations of the ACE2 protein were found where the maximum frequency of alteration was 3.49 %. On the other hand, 6 mutations at 4 different locations of the protein sequence were found for CXCL10 where the highest level of alteration frequency was 2.79% (Fig. 4). Mutations, amplifications, deletions, copy number alterations, and all other genetic changes are referred to as the functional significance of any gene in cancer development. Therefore, these results provided supportive evidence of the active participation of ACE2 and CXCL10 in lung cancer development.

For identifying and analyzing co-expressed genes of ACE2 and CXCL10 associated with lung cancer and COVID-19 we constructed two Venn diagrams by listing the co-expressed genes of ACE2 and CXCL10 in each case of lung cancer and COVID-19. A total of 3544 commonly co-expressed genes associated with both lung cancer and COVID-19 were identified for ACE2 whereas the number of the commonly co-expressed genes of CXCL10 was 2088. These commonly co-expressed genes were utilized to interpret the functional nature of ACE2 and CXCL10 both in lung cancer and COVID-19 development. By analyzing the molecular activity of the 3544 commonly co-expressed genes for ACE2, we found that 803 genes are involved in the binding activity (Fig. 7a and b). The active participation of these commonly co-expressed genes in binding activity can be beneficial to ACE2 for showing a more efficient binding affinity towards SARS CoV-2 viral spike protein. This functional enforcement of the receptor activity of ACE2 may increase the susceptibility of lung cancer patients towards COVID-19. On the other hand, to interpret the functional attitude of CXCL10 we used the list of 2088 commonly co-expressed genes of CXCL10 associated with lung cancer and COVID-19. By analyzing the biological activity of 2088 co-expressed genes, we found that 68 genes are directly involved in the immune system (Fig. 7c and d). At the early stage of our study, we showed overexpression of CXCL10 in lung cancer and by doing the functional analysis of CXCL10 we confirmed its association with immune response. By combining these two analyses, we can predict that overexpressed CXCL10 along with the presence of other cytokines causes cytokine storms in the alveoli of the COVID-19 infected lung cancer patients (Fig. 8). This cytokine storm results in the alveolar collapse due to lung cell damage by excessive neutrophil recruitment to the site of infection. In the prolonged severe condition, the damaged alveoli cause decreased gas exchange and result in difficulty in breathing. Finally, the ultimate destruction of the alveoli results in the obstruction of breathing and causes the patient's death (Szklarczyk et al., 2017, Mi et al., 2013). Overall, enforcing the viral entry and cytokine storm, the over-expressed ACE2 and CXCL10 become major factors of the higher susceptibility and fatality rate of lung cancer patients towards COVID-19 infection.

## 5 Conclusion

As biological processes are overly complex, analyzing a single signaling molecule or receptor in regards to a broad spectrum pathophysiological phenomenon triggered by different pathogens or diseases can raise concerns. However, the key findings of this research revealed some of the strong evidences confirming the direct interconnection of ACE2 and CXCL10 with lung cancer development. Therefore, based on this systemic analysis, we can pinpoint that ACE2 and CXCL10 are possible biomarkers whose higher expressions are responsible for the greater susceptibility and fatality of lung cancer patients towards the COVID-19 infection. This finding can facilitate in choosing drug targets for the management of COVID-19 through therapeutics and evaluating the type of measures that are required to be adapted for individuals with lung cancer. Although the in silico facts about the association of ACE2 and CXCL10 overexpression in lung cancer patients and COVID-19’s severe manifestations in these individuals coincide with peripheral relevant studies, further wet-lab experiments are required for in-depth understanding.

## CRediT authorship contribution statement

**Tousif Bin Mahmood:** Conceptualization, Methodology, Formal analysis, Writing – original draft. **Afrin Sultana Chowdhury:** Supervision, Writing – original draft, Writing – review & editing. **Mohammad Uzzal Hossain:** Validation, Writing – review & editing. **Mehedee Hasan:** Formal analysis, Writing – original draft. **Shagufta Mizan:** Writing – original draft, Writing – review & editing. **Md. Mezbah-Ul-Islam Aakil:** Formal analysis, Writing – original draft. **Mohammad Imran Hossan:** Writing – review & editing.

## Declaration of Competing Interest

The authors declare no competing interest.
